# High‐Conductivity Active Interface Layer for Active Lithium Reservoir in Micro‐Silicon‐Based All‐Solid‐State Batteries

**DOI:** 10.1002/advs.78198

**Published:** 2026-11-02

**Authors:** Ziqing Wang, Xinxin Zhu, Ruichen Zhu, Wendi Dou, Qian Zhang, Jun Lu, Junxiu Wu

**Affiliations:** ^1^ College of Chemical and Biological Engineering Zhejiang University Hangzhou China; ^2^ School of Chemistry and Materials Science Hangzhou Institute for Advanced Study University of Chinese Academy of Sciences Hangzhou China

**Keywords:** active interface layer, active lithium reservoir, all‐solid‐state lithium‐ion batteries, pre‐lithiation, silicon anodes

## Abstract

Sulfide‐based all‐solid‐state lithium‐ion batteries (ASSLIBs) employing fully active, carbon‐free micro‐silicon (µSi) anodes present significant potential for high energy density, enhanced safety, and reduced cost. However, their practical application is challenged by inherent issues such as sluggish reaction kinetics, substantial volume expansion, and low Coulombic efficiency, which collectively degrade overall electrochemical performance. Herein, an interfacial engineering strategy is developed to construct an active interface layer on µSi particles via a mechanochemical cold‐pressing method. The resulting Li─Si alloy layer acts as an active lithium reservoir, which enables active lithium reservoir. Furthermore, this spontaneously formed active layer exhibits high ionic/electronic conductivities and accommodates volume changes due to its favorable mechanical properties, thereby improving electrode reaction kinetics and structural stability. ASSLIBs fabricated with LiNi_0.83_Co_0.12_Mn_0.05_O_2_ cathode and modified µSi anode deliver a high initial specific capacity of 182.55 mAh g^−1^ and retain a capacity retention of 89.2% after 200 cycles at 0.1 C. This work offers a facile interfacial engineering technique for constructing self‐compensating lithium reservoirs and promotes the development of high‐energy‐density silicon‐based solid‐state batteries.

## Graphical abstract

A self‐generated Li–Si alloy interface transforms carbon‐free micro‐silicon into a kinetically accelerated and mechanically resilient anode for sulfide‐based all‐solid‐state batteries. Serving simultaneously as an active lithium reservoir and conductive interface, the alloy layer compensates lithium loss while accommodating volume changes, enabling 89.2% capacity retention over 200 cycles.

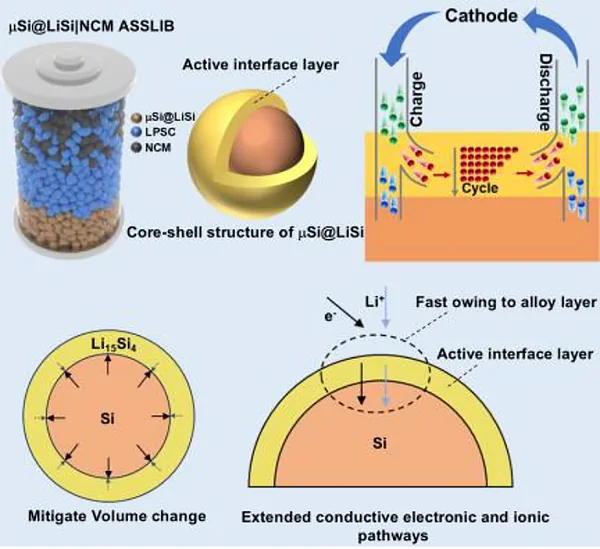

## 1 Introduction

All‐solid‐state lithium‐ion batteries (ASSLIBs) with sulfide electrolytes (SEs) are widely regarded as promising next‐generation energy storage technologies offering enhanced safety and higher energy density [1]. Among various anode materials, silicon anodes have attracted considerable interest owing to their high theoretical capacity (3579 mAh g^−1^) and moderate operating potential (∼0.4 V vs. Li^+^/Li) [2, 3, 4]. In particular, micro‐silicon (µSi) presents several practical benefits over nano‐silicon (nSi). Pure µSi can serve as the entire active anode material without requiring additional conductive or inactive additives, minimizing undesirable side reactions with SEs [5, 6]. This fully active electrode architecture maximizes the active‐material content, reduces the amount of inactive components, and simplifies the internal interfacial structure of the anode. It therefore offers potential advantages for improving both the gravimetric and volumetric energy densities of practical solid‐state batteries [7, 8, 9].

Despite these advantages, the interface between silicon and the rigid inorganic electrolyte remains susceptible to mechano‐chemical degradation. The substantial volume changes that silicon undergoes during cycling impair the mechanical integrity of the interface, resulting in crack formation that obstructs ionic conduction. Furthermore, sulfide electrolytes are thermodynamically prone to reduction at the silicon anode, generating electrically insulating and ionically blocking decomposition products. These side reactions increase interfacial resistance and further impede ion transport, resulting in chemically unstable solid electrolyte interphases (SEI) that markedly reduce Coulombic efficiency and accelerate capacity fading [10, 11, 12]. Conventional electrolyte modification strategies are insufficient to address the inherent reducibility of sulfide electrolytes [13, 14, 15, 16]. Therefore, targeted surface engineering of silicon is imperative for constructing interfaces compatible with sulfide‐based solid electrolytes.

In solid‐state systems, the absence of fluidity in solid electrolytes poses greater challenges in forming a stable electrolyte/electrode interface compared to liquid‐based systems. This necessitates the establishment of an interface that simultaneously exhibits high ionic and electronic conductivity, along with maintaining intimate physical contact with the electrolyte to support the reversible alloying process of silicon. To address these requirements, controlled pre‐lithiation on the silicon surface can be employed to form a lithium‐rich silicon (Li─Si) alloy layer [15, 17, 18, 19]. Li─Si alloys are known for their high ionic and electronic conductivities, which promote efficient Li^+^ transport across the interface [20]. Furthermore, the slight expansion associated with the Li─Si layer acts as a buffer during subsequent cycling, helping to maintain interfacial integrity between the silicon particles and the solid electrolyte [21]. The lithium within the alloy can also compensate for lithium loss during cycling, enabling a dynamic balance throughout lithiation and delithiation processes. These advantages contribute to improved initial Coulombic efficiency (ICE), enhanced reaction kinetics, and high interfacial stability. Therefore, the key to establishing stable mechanochemical interfaces lies in fabricating a thin and uniform alloy layer that integrates these beneficial characteristics.

In this work, we designed a homogeneous, electrochemically active Li─Si alloy layer on the surface of µSi particles (µSi@LiSi) through a pressure‐induced in situ pre‐lithiation strategy [22]. This alloy layer functions as an active lithium reservoir, effectively compensating for lithium loss during delithiation and facilitating uniform Li^+^ deposition during lithiation (Figure 1a). It also possesses a high affinity for Li and provides readily accessible Li‐containing sites, thereby lowering the kinetic barrier for Li insertion into the Si particles. The continuous alloy shell also enlarges the electrochemically active contact area, homogenizes the local Li‐ion flux, and facilitates the propagation of the lithiation reaction from the particle surface toward the Si core. Additionally, owing to the favorable mechanical properties of Li─Si alloy, the interlayer acts as a flexible buffer between µSi and the SEs, mitigating interfacial degradation from large volume changes and preserving intimate electrode/electrolyte contact, thereby substantially improving the mechanical integrity of the µSi anode [15]. More importantly, the Li─Si shell functions as a local mixed‐conducting interlayer that simultaneously facilitates electron transport and Li‐ion migration at the particle surface (Figure 1b) [23]. As a result, the full cell incorporating a LiNi_0.83_Co_0.12_Mn_0.05_O_2_ (NCM83) cathode and the µSi@LiSi anode delivered an initial specific capacity of 182.6 mAh g^−1^ with an ICE of 82.1%, and 89.22% capacity retention after 200 cycles at 0.1 C, with an average CE above 99.8%. Even at 0.5C, the µSi@LiSi anode retained 61.7% of its capacity after 1000 cycles. This work provides a facile interfacial engineering strategy for addressing µSi anode interfacial challenges in ASSLIBs. By constructing an active Li─Si alloy layer through simple pressure, it significantly enhances cycling stability and compatibility with SEs, presenting a practical and economical route toward next‐generation ASSLIBs with higher energy density and improved safety.

**FIGURE 1:**
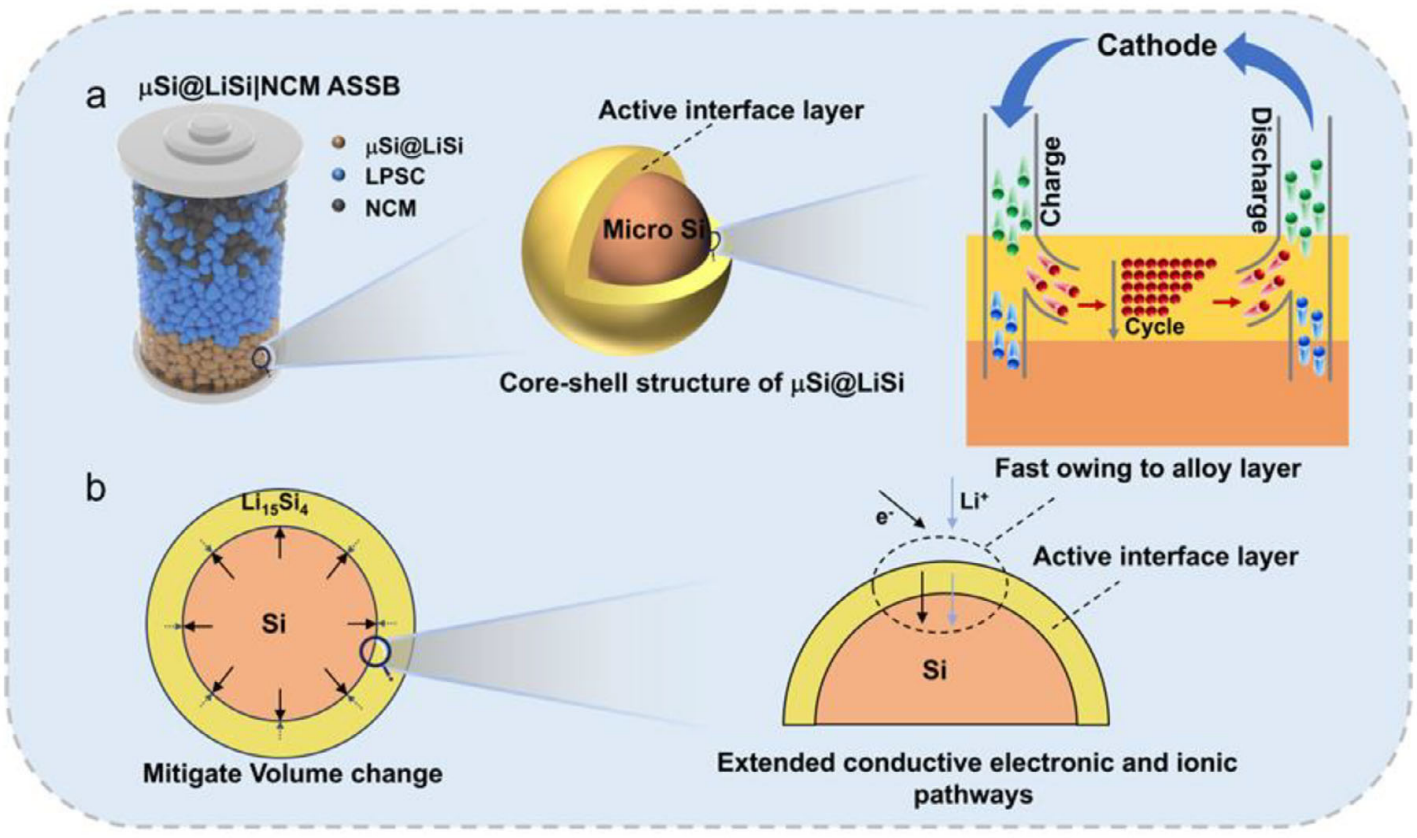
(a) Schematic of the µSi@LiSi full cells and the active lithium reservoir mechanism of the active interface layer. (b) Mechanism of active interface layer mitigating volume expansion and achieving high ionic/electronic conductivity.

## 2 Results and Discussion

### 2.1 In Situ Construction of Thin Li─Si Alloy Shell on µSi Particles

The µSi@LiSi core‐shell structure was synthesized through a mechanochemical cold‐pressing approach, driven by spontaneous interfacial reactions between Si particles and Li metal. As illustrated in Figure 2a, controlled pressure was applied to a stacked µSi layer and ultrathin lithium foil to trigger surface pre‐lithiation, followed by mechanical grinding to achieve uniform dispersion and form the final µSi@LiSi architecture. The cross‐sectional scanning electron microscopy (SEM) image in Figure 2b reveals a multilayer configuration consisting of a Cu current collector (∼10 µm), an intermediate ultrathin lithium layer (∼20 µm), and a µSi particle layer, consistent with EDS mapping results (Figure S1). Compared to pristine µSi particles, which display irregular morphologies and broad size distributions of 1–5 µm (Figure S2), the µSi@LiSi samples exhibit well‐defined polygonal boundaries and smoother surfaces (Figure 2c). To further investigate the surface structural evolution, transmission electron microscopy (TEM) and elemental mapping were systematically performed. As shown in Figure 2d, the µSi particles consist predominantly of elemental silicon, with only trace surface oxygen originating from the silicon oxide (SiO_x_) layer [24]. High‐resolution TEM (HRTEM) image of µSi particles reveals well‐defined lattice fringes with a spacing of 0.315 nm, corresponding to the (111) crystallographic plane of crystalline silicon (Figure 2e), alongside a ∼2 nm amorphous surface layer consistent with SiO_x_. In contrast, the µSi@LiSi materials exhibit a distinct core‐shell structure. Except for the similar lattice spacing of 0.314 nm for the (111) plane, a uniform and dense amorphous layer, approximately 5 nm thick, is visible on the particle surface, which is attributed to the formation of a Li─Si alloy interface layer (Figure 2g). It is worth noting that the high reactivity of the surface‐formed Li─Si alloys with atmospheric oxygen results in pronounced oxygen enrichment in Figure 2f. These comprehensive SEM and TEM characterizations demonstrate that lithium metal reacts preferentially with the silicon particles to form a Li─Si alloy interface layer, ultimately creating a well‐defined µSi@LiSi core‐shell structure. Meanwhile, to further substantiate the uniformity of this interphase, we performed TEM characterization on multiple Si particles from four regions (Figure S3), both large‐area elemental mapping and random spot analyses consistently confirm the presence of a continuous and uniform Li─Si alloy layer surrounding each Si microparticle, demonstrating the excellent reproducibility of our mechanochemical alloying strategy.

**FIGURE 2:**
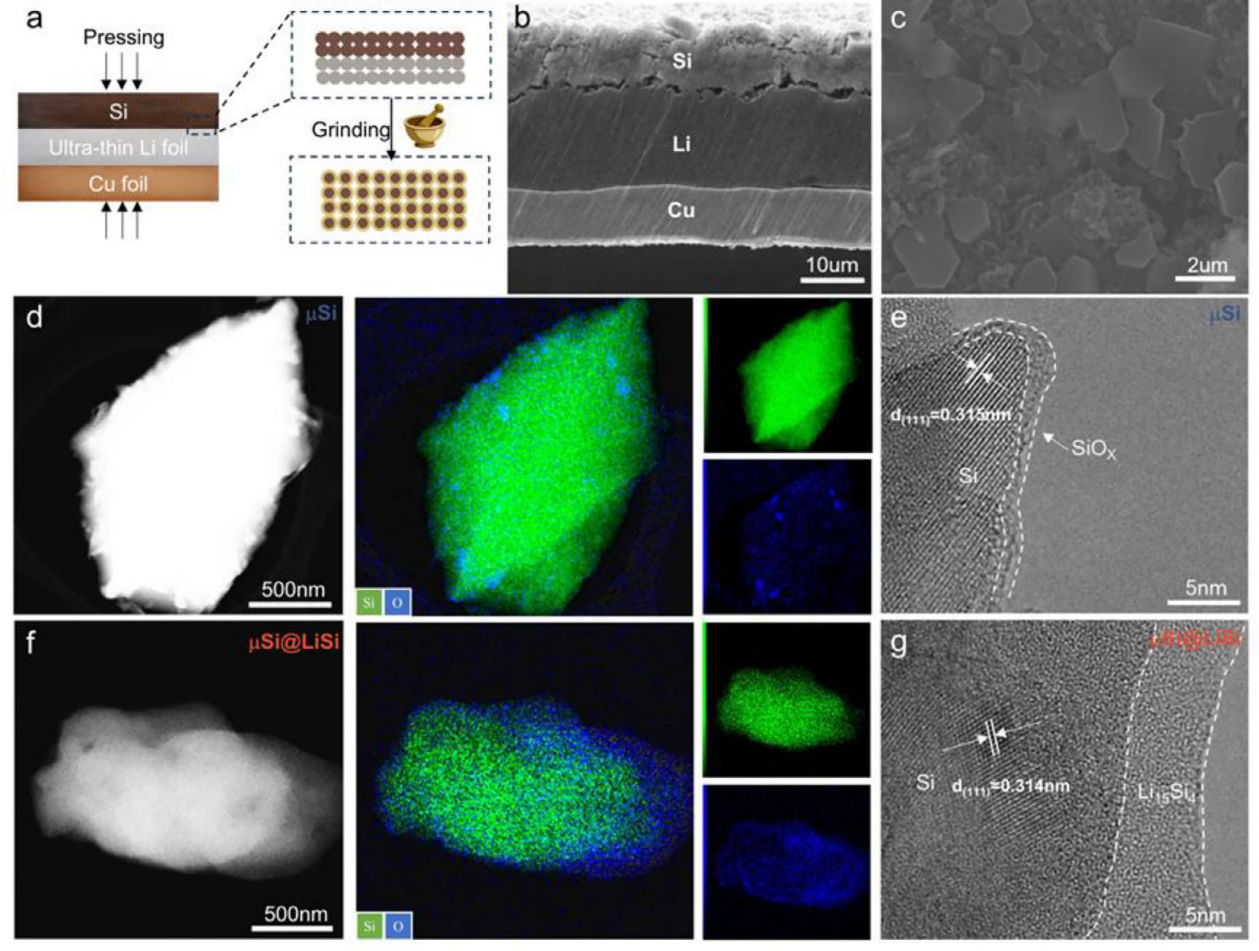
(a) Schematic of the in situ pre‐lithiation strategy of the core–shell structure µSi@LiSi anode. (b) Cross‐section SEM of µSi@LiSi anode before in situ pre‐lithiation. (c) SEM image of the µSi@LiSi particles. (d) TEM images and elemental mapping of µSi particles. (e) HRTEM images of the µSi particles. (f) TEM images and elemental mapping of µSi@LiSi particles. (g) HRTEM images of the µSi@LiSi particles.

To investigate the structural and compositional evolution of µSi and µSi@LiSi materials, x‐ray diffraction (XRD) analysis was performed (Figure 3a). Pristine µSi exhibited diffraction peaks at 28.4°, 47.3°, 56.1°, 69.1°, and 79.3°, corresponding to the (111), (220), (311), (400), and (331) planes of crystalline Si (PDF#03‐065‐1060). In contrast, µSi@LiSi retained the predominant crystalline silicon structure but additionally displayed distinct presence of the Li_15_Si_4_ phase, indicating the formation of Li─Si alloy after the mechanochemical cold‐pressing treatment. Raman spectroscopy further revealed a peak shift from 520.6 cm^−1^ for pristine µSi to 524.1 cm^−1^ for ¼Si@LiSi (Figure 3b). This noticeable blueshift indicates compressive strain within the inner silicon core, which originates from lattice distortions induced by the surrounding Li─Si alloy shell [25, 26]. To probe the chemical evolution process during µSi@LiSi preparation, solid state nuclear magnetic resonance (ssNMR) was conducted at different pressing times. ^7^Li ssNMR enables differentiation of the Li─Si alloy phase, which exhibits a broad signal centered around 0 ppm [27]. Figure 3c presents ^7^Li ssNMR spectra of µSi@LiSi samples pressed for 1 to 10 min, revealing clear time‐dependent structural changes. A remarkable ^7^Li signal at 0 ppm was observed in all pressed µSi@LiSi materials, corresponding to the Li─Si alloy phase. Crucially, the intensity of this signal increased with pressing time, indicating an increased fraction of Li─Si alloy formed. X‐ray photoelectron spectroscopy (XPS) provided complementary evidence of surface chemistry (Figure 3d). For µSi, the Si 2p spectrum featured characteristic Si─Si (99.0 eV) and Si─O (103.2 eV) bonds. In contrast, µSi@LiSi exhibited additional signals at 98.5 and 101.0 eV, assigned to Li─Si and Li─Si─O bonds, respectively, confirming the presence of a Li─Si alloy shell at the particle surface (Figure S4) [28, 29]. Depth profiling of µSi@LiSi further clarified the core‐shell distribution. As shown in Figure 3e, the initial surface spectra displayed attenuated Si 2p signals due to minor atmospheric oxidation during transfer, though Li─Si and Li─Si─O bonds were still detectable. With progressive etching, the Li─Si bond intensity initially exceeded the Si─Si component but was later surpassed with increasing depth, consistent with the trend of core‐shell architecture. Simultaneously, Li 1s spectra showed progressively decreasing intensity and lower binding energy toward deeper layers, corroborating the graded core‐shell architecture.

**FIGURE 3:**
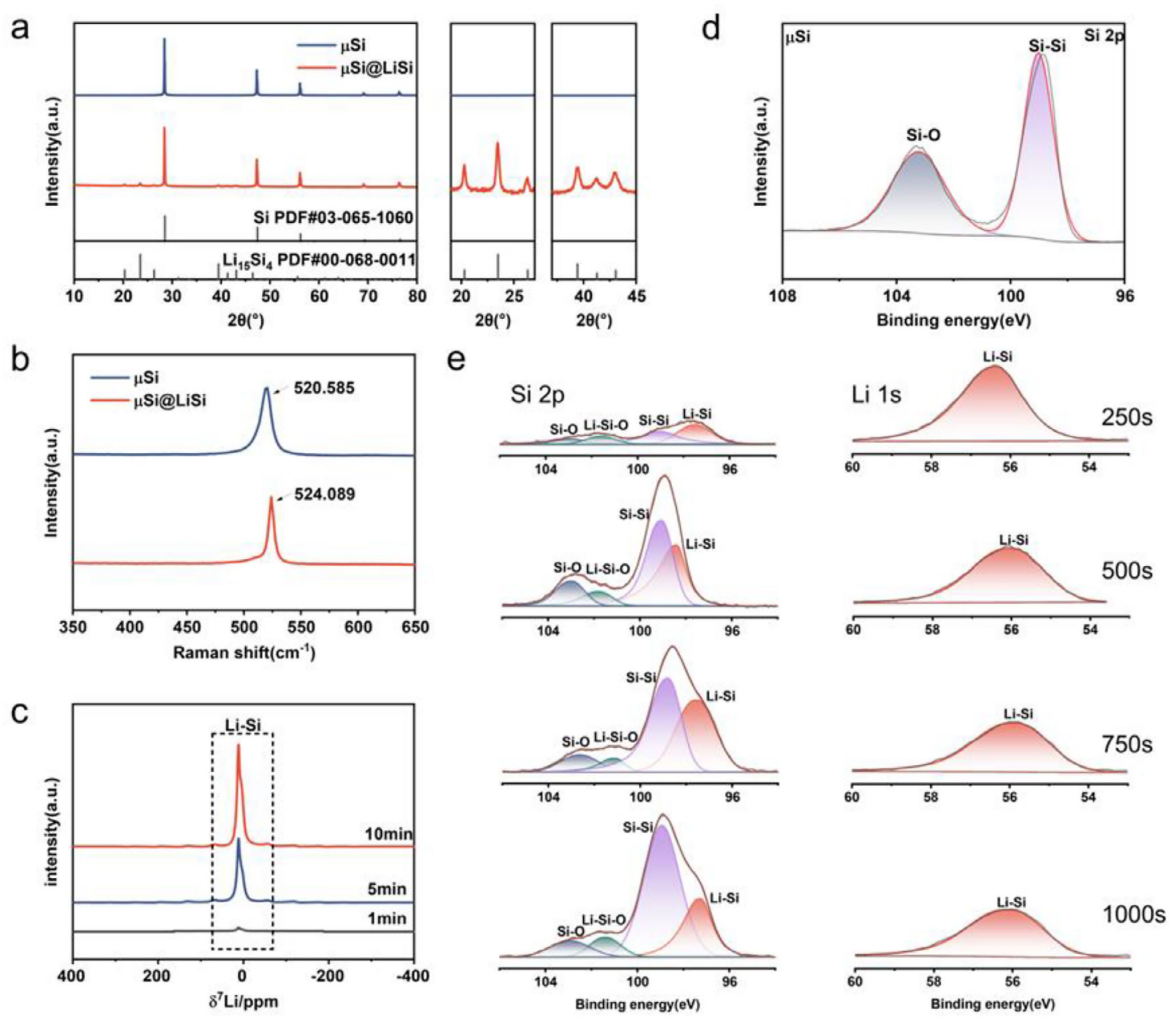
(a) XRD patterns of µSi and µSi@LiSi materials. (b) Raman spectrum of µSi and µSi@LiSi materials. (c) ^7^Li NMR spectra of µSi@LiSi materials with different pressure application times. (d) The XPS spectra of Si 2p in the µSi materials. (e) XPS depth profiling analysis of µSi@LiSi materials.

### 2.2 Reversible Electrochemical Activity of Li─Si Shell

To probe the electrochemical activity of Li in the µSi@LiSi anode, a µSi@LiSi||LPSC ||Li half‐cell was assembled with a galvanostatic charging process, as shown in Figure 4a. The µSi@LiSi electrode contains 0.15 mg of Li (determined by the mass difference between the Li‐Cu composite and the Cu current collector), with a mass ratio of Li to Si of approximately 1:10. Thus, the theoretical capacity of the Li in the electrode is calculated to be 0.579 mAh (considering that lithium has a theoretical capacity of 3860 mAh g^−1^), while the actual capacity is 0.57 mAh, confirming that Li^+^ can be extracted from the Li─Si alloy shell. This demonstrates that the surface alloy layer is highly electrochemically active, enabling Li release that enhances both ICE and reversible capacity. To probe the electrochemical reversibility and cycling stability, symmetric cells with a µSi@LiSi||LPSC||µSi@LiSi configuration were assembled. The galvanostatic Li plating/stripping profiles (Figure 4b) reveal the exceptional long‐term stability of the µSi@LiSi electrodes. Under conditions of 0.2 mA cm^−2^ and 0.2 mAh cm^−2^, the cell demonstrated exceptional stability over 4000 h, with the overpotential increasing only from 200 to 250 mV (Figure S5). This extended cycling stability demonstrates that the Li─Si interfacial shell effectively regulates the uniform deposition of lithium and potentially enables active lithium reservoir to the µSi core, thereby serving as an active lithium reservoir to continuously supplying active lithium. Furthermore, the current‐density stability was tested, which reflects the capability of the electrode to maintain stable alloying/dealloying reactions without rapid interfacial degradation or cell failure. As depicted in Figure 4c, the µSi@LiSi symmetrical cell remained electrochemically stable even at current densities up to 1.6 mA cm^−2^, indicating significantly favorable interfacial stability and charge‐transfer kinetics. It should be noted that, for this symmetric‐cell test, the lithium loading was increased to approximately 0.22 mg, corresponding to a capacity of about 0.85 mAh, to ensure a sufficient lithium inventory for evaluating the lithiation/alloying capability of the µSi@LiSi anode and the associated interfacial transport behavior at higher current densities. Thus, the µSi@LiSi anode exhibits excellent alloying stability, reducing the risk of dendrite formation compared to the µSi anode (Figure 4d).

**FIGURE 4:**
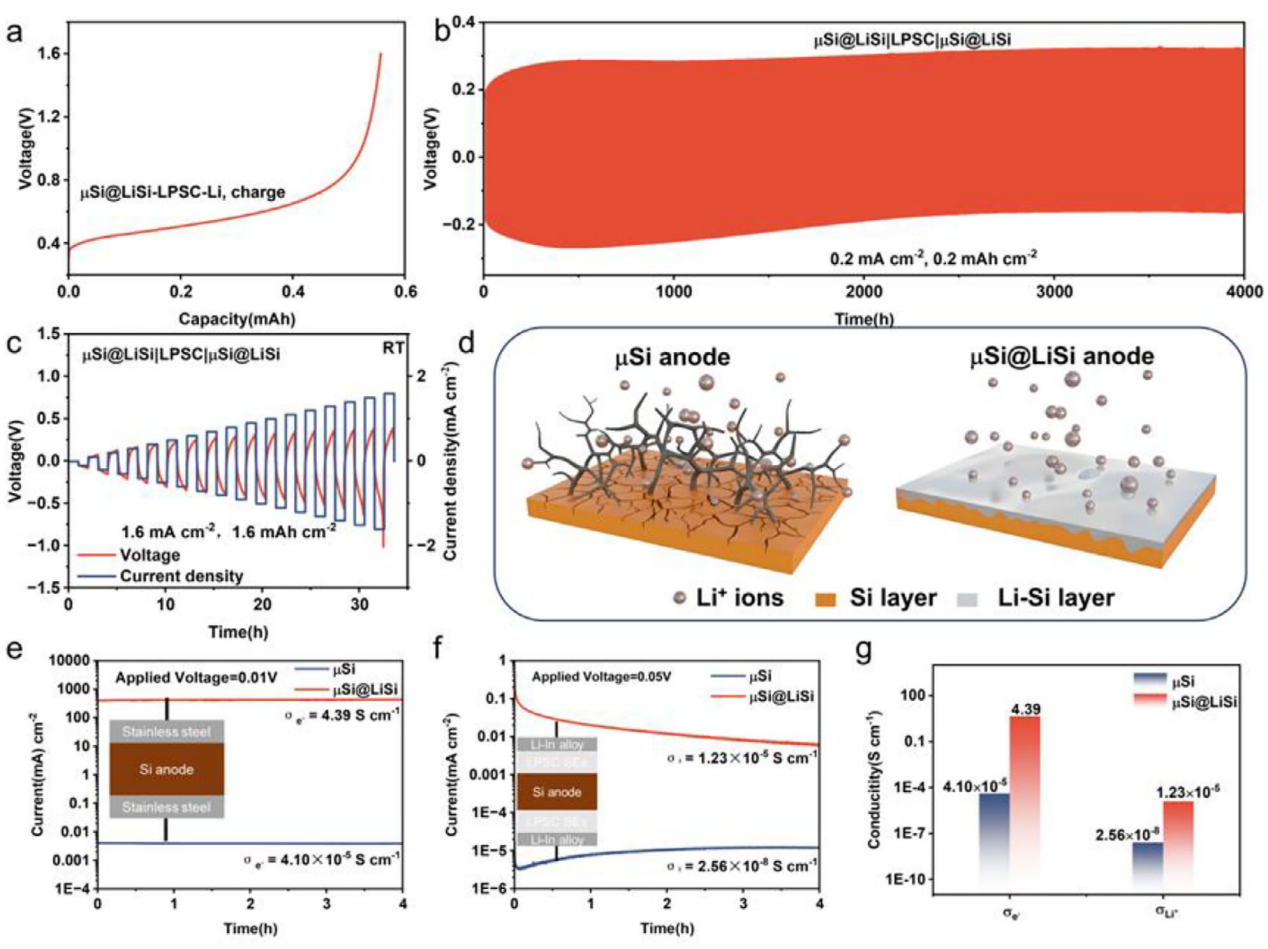
(a) Charging test of µSi@LiSi||LPSC||Li half cell. (b) Discharge–charge profiles of µSi@LiSi||LPSC|| µSi@LiSi symmetric cell, recording under operating conditions of 0.2 mA cm^−2^. (c) current‐density stability testing of the µSi@LiSi||LPSC||µSi@LiSi symmetric cell, conducted with a fixed Li plating/stripping time of 1 h. (d) Schematic of the active interface layer in µSi@LiSi promoting uniform lithium deposition. (e) Electronic partial conductivity determination of µSi and µSi@LiSi anodes based on DC polarization measurements. (f) Ion partial conductivity determination of µSi and µSi@LiSi anode based on DC polarization measurements. (g) Comparison of ionic and electronic conductivity of µSi and µSi@LiSi anodes.

Meanwhile, the construction of the active interface Li─Si layer in µSi@LiSi is conducive to improving the electrical and ionic transport. To quantify the ionic and electronic conductivities of µSi and µSi@LiSi materials, the DC polarization method based on symmetrical cells with ion‐blocking or electron‐blocking electrodes was employed. A three‐layered symmetric SS||µSi@LiSi||SS cell was assembled to measure the electronic conductivity, as depicted in the inset of Figure 4e. Based on the results from the dependence of polarization voltages on response currents, it can be concluded that the resistance of the SS||µSi||SS and SS||µSi@LiSi||SS cells is 5168 and 0.047 Ω, respectively. Given a thickness (0.104 cm) and area (0.5 cm^−2^) for the tested pellet, the electronic conductivities (σ_e_) of µSi and µSi@LiSi were calculated to be 4.10 × 10^−5^ and 4.39 S cm^−1^. Conversely, the ionic conductivities of µSi and µSi@LiSi were respectively measured with the SS||Li‐In||SE||µSi||SE||Li‐In||SS and SS||Li‐In||SE||µSi@LiSi||SE||Li‐In||SS symmetrical cells (Figure 4f), resulting in ionic conductivities of 2.56 × 10^−8^ and 1.23 × 10^−5^ S cm^−1^. Importantly, for µSi@LiSi, these values depend on the Li content and the resulting Li─Si phase fraction. Increasing the Li content promotes the formation of a larger amount of the Li─Si alloy phase, which provides more favorable mixed ionic/electronic transport than pristine Si. The lithium content here is 0.15 mg. As shown in Figure 4g, the µSi@LiSi anode exhibits favorable performance in both electronic conductivity and ionic conductivity compared to µSi. This enhancement confirms that the µSi@LiSi anode functions as a superior mixed ionic‐electronic conductor, attributed to the electrochemical properties of the active interface Li─Si alloy layer. Therefore, the surface‐formed Li─Si alloy should be regarded as a dynamic active interphase rather than a permanently fixed shell. Although the Li─Si alloy is thermodynamically driven to further alloy with the Si core during repeated cycling, its electrochemical function is continuously renewed through reversible alloying/dealloying reactions. Consequently, the interface evolves dynamically while preserving rapid ionic/electronic transport.

### 2.3 Electrochemical Performance

To evaluate the effect of active Li─Si interface layer, full cells with NCM83 cathodes and either µSi@LiSi or µSi anodes were assembled. Figure 5a shows the initial galvanostatic charge/discharge profiles at 0.1 C with a negative/positive capacity ratio of 1.2. The µSi@LiSi anode delivered an initial discharge capacity of 182.6 mAh g^−1^ with an ICE of 82.1%, compared to 179.0 mAh g^−1^ and 76.9% for the µSi anode. The enhanced ICE of the µSi@LiSi anode demonstrates the positive role of the Li─Si alloy layer in improving active material utilization and enabling active lithium reservoir. In addition, the polarization of the µSi@LiSi anode is significantly lower than that of µSi, attributed to enhanced conductivity and improved electrode/electrolyte contact facilitated by the active interface layer. Differential capacity (dQ/dV) analysis (Figure 5b and Figures S6 and S7) further confirmed these advantages. For the µSi@LiSi | NCM83 cell, three oxidation peaks were observed at 3.50–3.67, 3.90, and 4.10 V, corresponding to the H1→M, M→H2, and H2→H3 phase transitions of NCM83, respectively, with fully reversible reduction peaks [30]. Compared with µSi, the µSi@LiSi anode showed reduced polarization, higher voltage, and stronger peak intensities, indicative of accelerated reaction kinetics and higher reversible capacity. Furthermore, long‐term cycling stability tests were conducted at 0.1 C (Figure 5c). Figure S8 displays the initial activation process of µSi and µSi@LiSi anodes. The µSi@LiSi | SE | NCM83 full cell demonstrated outstanding cycling stability, achieving an initial discharge capacity of 182.6 mAh g^−1^ with an ICE of 82.1%, and retaining 89.2% capacity after 200 cycles with an average CE of 99.8%. In contrast, the µSi | SE | NCM83 cell retained only 49.7% after 50 cycles. These results highlight that the Li─Si shell enables active lithium reservoir and effective volume buffering, whereas µSi suffers rapid capacity fading due to lithium loss and interfacial instability. Even under higher current density at 0.5C, the µSi@LiSi anode maintained 61.7% capacity retention after 1000 cycles (Figure 5d). The µSi@LiSi anode also exhibited outstanding rate performance (Figure 5e,f). Specifically, the µSi@LiSi anode delivered discharge capacities of 166.5, 148.7, 121.3, and 96.3 mAh g^−1^ at 0.1C, 0.2C, 0.5C, and 1C, respectively. As the rate returned to 0.1 C, the discharge capacity recovered to 165.5 mAh g^−1^, confirming high rate performance. In comparison, the µSi anode exhibited much poorer rate performance (Figure S9), primarily due to insufficient conductivity and mechanical stability. In Figure 5g, a comparison of the electrochemical performance in this study is provided in conjunction with other reported Si‐based sulfide ASSLIBs. After comprehensively considering parameters such as safety, environmental friendliness, scale‐up potential, preparation efficiency, capacity retention, and cycle numbers, the µSi@LiSi anode demonstrated in our work is currently the leading sulfide ASSLIB using µSi at room temperature. Table S1 compares the long‐term cycle stability of the µSi@LiSi | NCM83 cell with reported ASSLIBs with areal capacity higher than 1 mAh cm^−2^. It can be concluded that the µSi@LiSi anode achieves advanced levels of capacity retention, cycle lifespan, and ICE.

**FIGURE 5:**
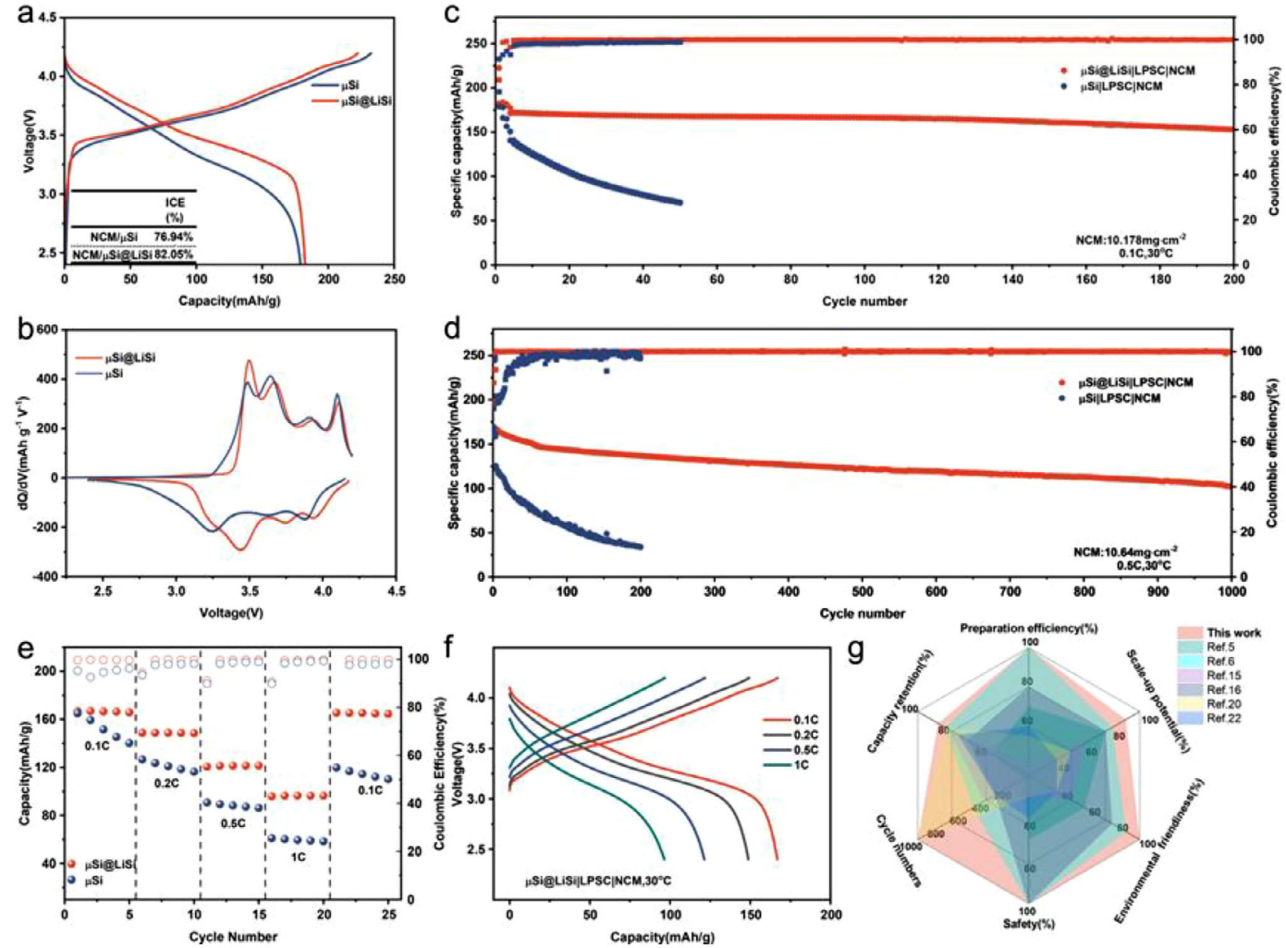
Electrochemical performance of µSi / µSi@LiSi | NCM83 full cells. (a) Voltage profiles. (b) dQ/dV profiles. (c) Long‐term cycling stability at 0.1 C. (d) Long‐term cycling stability at 0.5C. (e) Rate capability. (f) Charge/discharge curves at different rates. (g) Comparison of the advancement of µSi@LiSi | NCM83 cell with reported ASSLIBs with areal capacity higher than 1 mAh cm^−2^.

### 2.4 Electrochemical Kinetics Analysis

The evolution of interfacial impedance was evaluated by electrochemical impedance spectroscopy (EIS) measurements. Figure S10 depicts the Nyquist plots of µSi@LiSi and µSi cells before and after cycling. Before cycling, the µSi@LiSi anode exhibited a markedly lower charge‐transfer resistance (*R*_ct_, 23.4 Ω) compared with µSi (420.7 Ω). After cycling, the *R*_ct_ of both anodes increased to 305.2 and 895.8 Ω, indicating a robust interface between the µSi@LiSi anode and electrolyte. To further evaluate the resistance evolution during operation, in situ EIS was employed at different charge‐discharge states (Figure 6a,b). During the first cycle (Figure 6c,e,g,i), µSi@LiSi consistently displayed smaller impedance variations than µSi, reflecting superior conductivity and faster reaction kinetics. To distinguish the various resistance components, the impedance spectra were further assessed through the distribution of relaxation times (DRT) analysis to reveal the mechanism of interfacial properties on the electrochemical behavior of ASSLIBs (Figure 6d,f,h,j) [31]. Because NCM83/LPSC/µSi and NCM83/LPSC/µSi@LiSi full cells employed identical cathode compositions and LPSC electrolyte layers, the differences in their impedance responses can be primarily attributed to the anode side, particularly the intrinsic transport properties of the anodes and the anode/electrolyte interfaces. At the initial state, DRT profiles (Figure S11) depicted the similar impedance differences observed in Nyquist plots. During cycling, the bulk resistance (*R*_LPSC_) remained essentially unchanged, primarily arising from the LPSC layer, as indicated by the consistent peak located in 10^−5^ s [32]. Peaks in the τ range of 10^−4^‐0.1 s are mainly associated with ion transport across the electrode/electrolyte interphases, including contributions from the SEI and CEI, exhibiting stable intensity owing to the stabilized electrode structure and uniform SEI formation [33, 34]. Peaks in the τ range of 0.1–10 s, associated with interfacial charge transfer, further corroborated enhanced kinetics in the µSi@LiSi anode, attributed to superior electrode‐electrolyte contact. The peak of d/c with the largest time constants was substantially affected by the lithiation/delithiation states, which can be attributed to the ion diffusion or capacitive behavior of the ASSLIBs [35]. The low peak intensity of the µSi@LiSi anode was primarily due to its consistently higher lithiation state, as well as the improved interfacial conductivity and structural integrity. Notably, similar DRT trends were observed in the second cycle (Figures S12 and S13), indicating that active lithium reservoir remains effective during subsequent cycles. In a word, in situ EIS and DRT confirmed that the active interface layer in µSi@LiSi anode enhances the reaction kinetics and ensures robust solid‐solid interfaces, directly contributing to its superior capacity retention and rate capability. To further investigate differences in electrochemical kinetics between µSi and µSi@LiSi anodes, the *D*_Li_^+^ values for the two electrodes were measured by galvanostatic intermittent titration technique (GITT). As shown in Figure 6k,l and Figure S14a, the µSi@LiSi anode showed higher *D*_Li_^+^ (1.7 × 10^−9^ cm^2^ s^−1^) than the µSi anode (7.9 × 10^−10^ cm^2^ s^−1^). Consistent results were also observed in the GITT measurements of half‐cells (Figure S14b), which further confirmed the superior kinetics of the µSi@LiSi anode. This improvement arises from the Li─Si interfacial layer, which enhances both mechanical compatibility and ionic conductivity at the solid‐solid interface, corresponding to the results of EIS tests.

**FIGURE 6:**
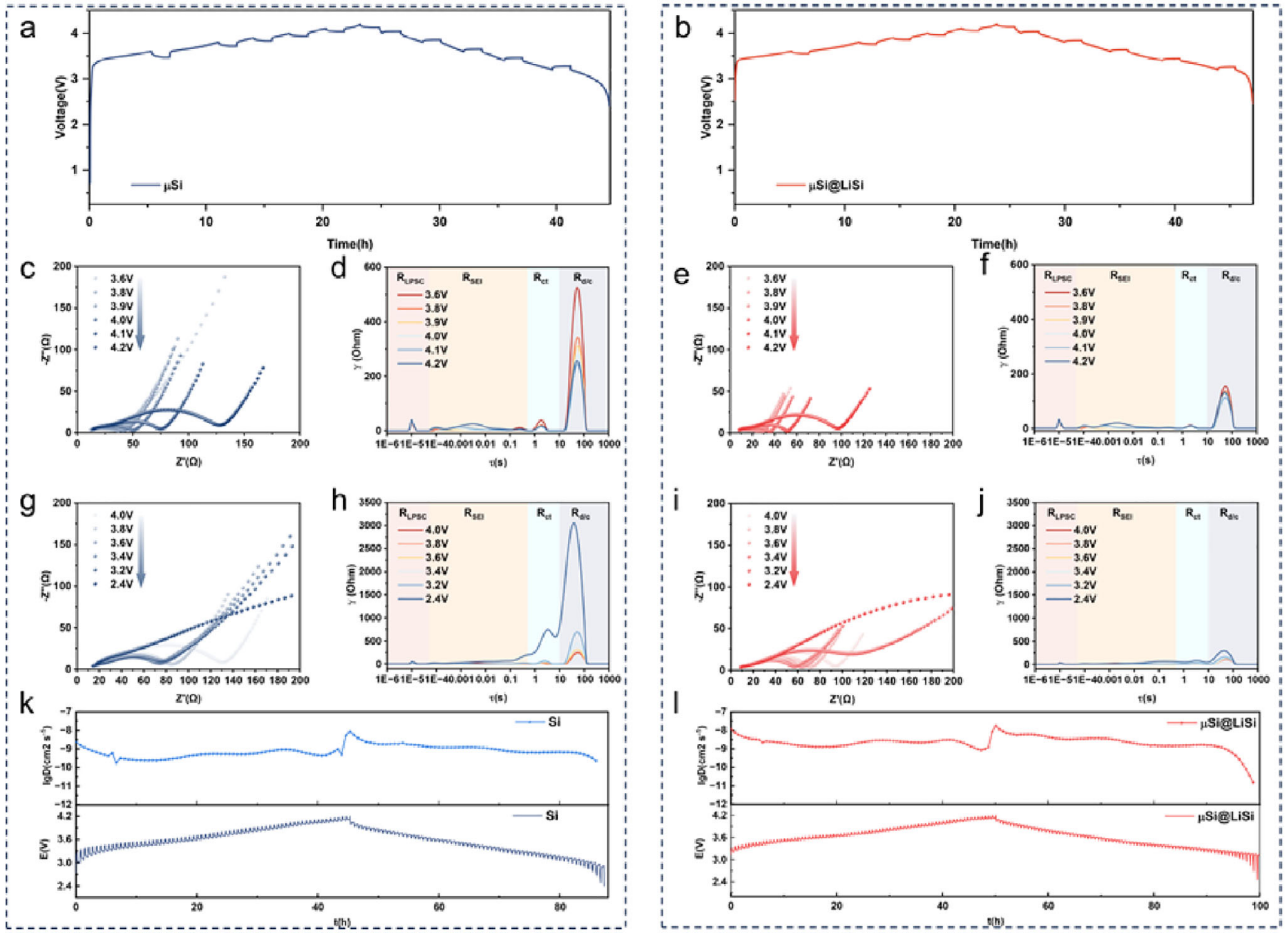
The initial voltage‐time curves of (a) the µSi and (b) µSi@LiSi anode. EIS spectra and DRT fitting results of different (c,d) charge and (g,h) discharge depths using the µSi anode. EIS spectra and DRT fitting results of different (e,f) charge and (i,j) discharge depths using µSi@LiSi anode. GITT curves and apparent *D*_Li_^+^ values of (k) µSi and (l) µSi@LiSi anodes based on full cells.

### 2.5 Analysis of Interface Chemo‐Mechanical Stability

To assess the morphological evolution of µSi and µSi@LiSi anodes, SEM images of the µSi and µSi@LiSi electrodes before and after cycling were acquired. Top‐view SEM images (Figure 7a,b) showed that pristine µSi electrodes consisted of loosely stacked particles with abundant voids, whereas µSi@LiSi electrodes formed a compact architecture with minimal voids, attributed to the robust mechanical properties of the Li─Si alloy shell. After cycling, the µSi particles underwent electrochemical sintering, coalescing into a dense and continuous film where the original particle boundaries became indiscernible (Figure 7c,d and Figure S15) [21]. However, this film exhibited severe cracking induced by the excessive volume expansion of silicon. These cracks disrupted the pathways for ion and electron transport and compromised the electrode‐electrolyte interface, ultimately leading to cell failure. By contrast, the µSi@LiSi anode preserved a stable architecture, as the alloy interface layer buffered stress and accommodated intrinsic Si expansion, thereby maintaining both structural and electrochemical stability. Figure 7e,f and Figure S16 showed the cross‐sectional SEM images and elemental mapping of both anodes. The µSi anode exhibited vertical cracks traversing the electrode, whereas the µSi@LiSi anode maintained a coherent structure. Atomic force microscopy (AFM) confirmed this trend. After 100 cycles, the average surface roughness of µSi increased to 513 nm, while µSi@LiSi maintained a much smoother surface of 246 nm, consistent with its superior mechanical integrity (Figure 7g,h). To further evaluate the interfacial stability of the electrodes after cycling, XPS analysis was conducted on both electrodes (Figure 7i–k). In the P 2p spectrum of the µSi electrode, strong signals of Li_3_P (130.3 eV) and Li_2_S (160.5 eV), along with PS_4_^3−^ species from the LPSC electrolyte (131.7 and 132.6 eV), indicated significant electrolyte decomposition and poor interfacial compatibility. In contrast, only trace amounts of Li_2_S and Li_3_P were detected in µSi@LiSi, confirming that the active interface layer effectively suppresses parasitic side reactions and stabilizes the solid‐solid interface. To further probe the interfacial evolution after prolonged cycling, XPS depth‐profiling measurements were performed on the µSi and µSi@LiSi anodes after 100 cycles (Figures S17 and S18). For both electrodes, the major interphase species show relatively small variations with increasing sputtering time, indicating a comparatively uniform interphase composition within the analyzed depth. Notably, the µSi@LiSi electrode exhibits a relatively stronger Li‐related signal and lower contributions from decomposition products such as Li_2_S and Li_3_P compared with the pristine µSi electrode. These results suggest that the µSi@LiSi anode retains a more Li‐rich interfacial environment after prolonged cycling while suppressing excessive interfacial decomposition. Such an interfacial composition is consistent with the role of the preformed Li─Si alloy layer as an active lithium reservoir and may facilitate Li‐ion transport across the anode/electrolyte interface. These results indicate that a dual‐functional active Li─Si interface layer was successfully established through in situ pre‐lithiation, which not only enables active lithium reservoir but also maintains the structural integrity of the anode, thereby avoiding particle pulverization and interface cracks that can lead to chemo‐mechanical failure.

**FIGURE 7:**
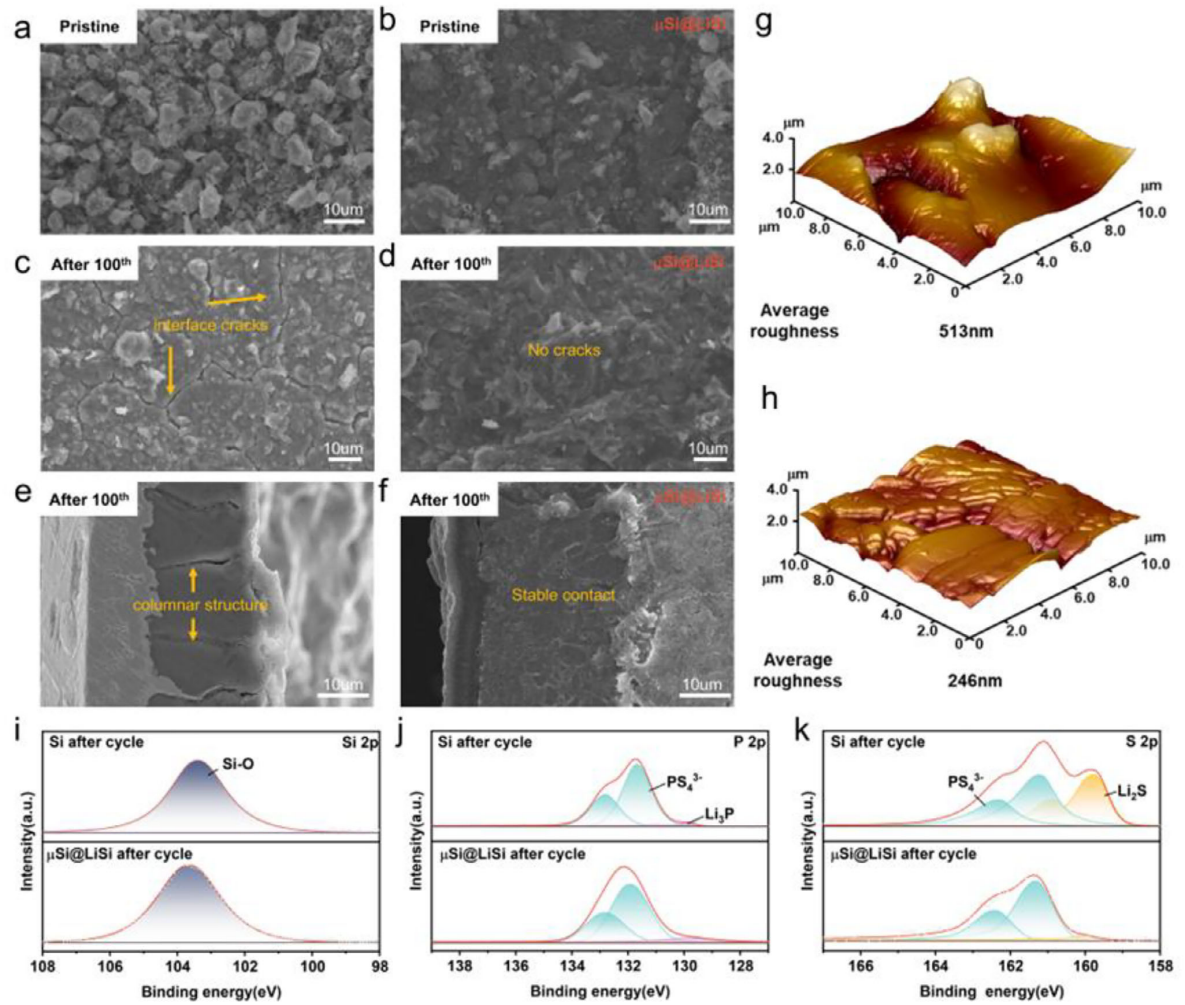
(a,b) The top‐view SEM images of µSi and µSi@LiSi anodes before cycling. (c,d) The top‐view SEM images of µSi and µSi@LiSi anodes after 100 cycles. (e,f) The cross‐section SEM images of µSi and µSi@LiSi anodes after 100 cycles. (g,h) AFM images of the surface morphology of µSi and µSi@LiSi electrodes in full cells after 100 cycles. (i–k) The XPS spectra of Si 2p, P 2p, and S 2p in the µSi and µSi@LiSi materials.

## 3 Conclusion

In summary, we have successfully synthesized a µSi@LiSi anode with a core‐shell structure, comprising a µSi core and a Li─Si alloy shell, through a mechanochemical cold‐pressing process driven by spontaneous interfacial reactions between µSi particles and lithium metal. The Li─Si alloy shell exhibits high ionic and electronic conductivity and serves as an active lithium reservoir, effectively compensating for lithium loss during cycling. Its favorable mechanical properties also act as a buffer layer that accommodates volume variations of the µSi core, thereby helping to maintain the structural integrity of the anode. Benefiting from these attributes, the µSi@LiSi anode delivers exceptional performance when integrated into sulfide‐based ASSLIBs with an NCM83 cathode, retaining 89.2% of its capacity after 200 cycles at 0.1 C and 61.7% after 1000 cycles at 0.5C, significantly outperforming conventional silicon anodes. This study demonstrates a facile interfacial engineering strategy for constructing self‐compensating lithium reservoirs, providing a practical pathway toward high‐energy‐density and long‐cycle‐life ASSLIBs.

## 4 Experimental Section

### 4.1 Materials Preparation

Li_6_PS_5_Cl (LPSC) was prepared by mechanically ball milling a stoichiometric mixture of Li_2_S (Sigma, 99.9%), P_2_S_5_ (Macklin, 99%), and LiCl (Aladdin, 99.9%) for 20 h at 600 r/min. Then, sintering at 550 °C for 25 h in a sealed quartz tube. All preparation processes were performed in an argon‐filled glovebox. The silicon particles were purchased from Alfa Aesar. LiNi_0.83_Co_0.12_Mn_0.05_O_2_ (NCM83) was purchased from Gauss Lithium Battery Co., Ltd. Lithium‐copper composite strip with ultra‐thin Li foil (20 µm) was purchased from Tianjin Zhongneng Lithium Co., Ltd. The copper current collector was purchased from Canrd Co., Ltd.

### 4.2 Electrode Preparation

The µSi@LiSi particles are prepared by mechanically cold pressing ultrathin Li foil and Si particles under 100 MPa pressure, followed by grinding for 0.5 h. To prepare the composite cathode, NCM83 and LPSC were milled in a mortar for 0.5 h with a weight ratio of 70:30. Both anodes and cathodes have a diameter of 8 mm. All procedures were performed in an argon atmosphere glove box (O_2_ < 0.1 ppm, H_2_O < 0.1 ppm).

### 4.3 Materials Characterization

X‐ray diffraction (XRD; Rigaku, SmartLab) was conducted using Cu Kα radiation over a 2θ range of 10°–80° with a step size of 10° min^−1^. Samples were sealed in an argon atmosphere using a quartz glass XRD sample holder and cover film (sealing tape). The surface and cross‐sectional morphology of the samples was characterized using a field‐emission scanning electron microscope (SEM; HITACHI, SU‐8100) with an energy‐dispersive x‐ray detector (EDX; Oxford, X‐max80). The composition and morphology of samples were characterized by transmission electron microscopy (TEM; JEOL, JEM‐F200); x‐ray photoelectron spectroscopy (XPS; Thermo Scientific, K‐Alpha+) with monochromatic Al Kα radiation was used to study the chemical composition of the µSi@LiSi anode materials. Raman tests were performed with a LabRAM Odyssey (Horiba) spectrometer running at a wavelength of 532 nm to characterize the structure of materials. The preparation process of Li─Si alloy was characterized by Solid State Nuclear Magnetic Resonance (ssNMR; Bruker, 600 MHz Avance III HD). Atomic force microscopy (AFM) test and average roughness of the electrode surface were measured on a Bruker Dimension Icon AFM

### 4.4 Cell Fabrication

To fabricate symmetrical cells, 130 mg of Li_6_PS_5_Cl (SE) was first pressed between two stainless steel rods at 220 MPa in the polyether ether ketone (PEEK) mold, resulting in a 10 mm diameter SE layer. Then, µSi@LiSi powders and two pieces of Cu foil were pressed on both sides of the LPSC pellet to form a sandwich structure at 110 MPa.

To fabricate full cells, 200 mg of Li_6_PS_5_Cl was first pressed at 220 MPa to form a pellet with a diameter of 15 mm. A Composite NCM83 cathode was put on one side of the SE pellet, and the other side was placed with the µSi@LiSi anode in turn (or Si anode). Finally, the pellet was formed by pressing at 220 MPa for 10 min. Subsequently, all assembled cells were tested under a constant stack pressure of 100 MPa to ensure intimate contact.

### 4.5 Electrochemical Measurements

Galvanostatic charge‐discharge was conducted in a voltage range of 2.4‐4.2 V via a battery test system (NEWARE, CT‐4008T) at 30 °C. The N/P ratio is 1.2 (based on the capacity of anode is 2500mAh/g, the capacity of cathode is 175 mAh/g, the anode loading of 0.85 mg/cm^2^ and cathode loading of 10.20 mg/cm^2^). Electrochemical impedance spectroscopy (EIS) measurements were performed on an electrochemical workstation (Biologic). The distribution of relaxation time (DRT) was completed using the MATLAB tools, as referred to in Zhang et al. [31]. Galvanostatic intermittent titration technique (GITT) tests for full cells were conducted on a NEWARE battery test system (CT‐4008T). Electronic or ionic partial conductivities of materials were determined based on a DC polarization method (Biologic).

## Author Contributions

**Ziqing Wang**: writing – original draft. **Xinxin Zhu**: validation. **Ruichen Zhu**: visualization. **Wendi Dou**: project administration. **Qian Zhang**: formal analysis. **Jun Lu**: writing – review and editing. **Junxiu Wu**: conceptualization, writing – review and editing.

## Conflicts of Interest

The authors declare no conflicts of interest.

## Supporting information




**Supporting File**: advs78198‐sup‐0001‐SuppMat.docx.

## Data Availability

The data that support the findings of this study are available from the corresponding author upon reasonable request.
