# Supplementary material for: High‐Conductivity Active Interface Layer for Active Lithium Reservoir in Micro‐Silicon‐Based All‐Solid‐State Batteries
Source: Adv Sci (Weinh). 2026 Nov 2:e78198. Online ahead of print. doi: 10.1002/advs.78198 (PMC13725741; doi:10.1002/advs.78198)
Supplement: Supplementary file 1 [file ADVS-9999-e78198-s001.docx]

**Supplementary information**

**High-Conductivity Active Interface Layer for Active Lithium Reservoir in Micro-Silicon-Based All-Solid-State Batteries**

*Ziqing Wang**^1^, Xinxin Zhu^1^*, Ruichen Zhu^1^, Wendi Dou^1^, Qian Zhang^1^, Jun Lu^1^*,* *Junxiu Wu^2^**

^1^College of Chemical and Biological Engineering, Zhejiang University, Hangzhou 310058, China

^2^ School of Chemistry and Materials Science, Hangzhou Institute for Advanced Study, University of Chinese Academy of Sciences, Hangzhou 310024, China

*Corresponding authors: [zhuxinxin@zju.edu.cn](mailto:zhuxinxin@zju.edu.cn); [junzoelu@zju.edu.cn](mailto:junzoelu@zju.edu.cn);

[wujunxiu@zju.edu.cn](mailto:wujunxiu@zju.edu.cn)

**Figure S1.** Cross-section SEM of μSi@LiSi anode before *in-situ* pre-lithiation and corresponding EDS mapping images.

**Figure S2.** SEM of μSi particles at different scales.

**Figure S3.** a-d) The TEM elemental mapping of different μSi@LiSi particles.

**Figure S4.** a) Si 2p XPS spectra of the initial μSi@LiSi anode sheet. b) Li 1s XPS spectra of the initial μSi@LiSi anode sheet.

**Figure S5.** Discharge-charge profiles of μSi@LiSi | LPSC | μSi@LiSi symmetric cells at 1000, 2000, 3000 cycles.

**Figure S6.** The dQ/dV curves of the μSi | NCM cell at 0.05 C.

**Figure S7.** The dQ/dV curves of the μSi@LiSi | NCM cell at 0.05 C.

**Figure S8.** a) The activation of the μSi | LPSC | NCM cell at 0.05 C. b) The activation of the μSi@LiSi | LPSC | NCM cell at 0.05 C.

**Figure S9.** Charge-discharge curves of the μSi | SE | NCM cell at different C rates.

**Figure S10.** Nyquist plots and corresponding fitting curves of μSi and μSi@LiSi anodes a) before and b) after cycling.

**Figure S11.** DRT fitting curves of two anodes before cycling.

**Fig. S12.** DRT fitting results of different a) charge and b) discharge depths using μSi anode during the second cycle.

**Figure S13.** DRT fitting results of different a) charge and b) discharge depths using μSi@LiSi anode during the second cycle.

**Figure S14.** GITT curves and apparent *D*_Li_^+^ values of μSi and μSi@LiSi anode based on a) full cells and b) half cells (*vs.* Li-In).

**Figure S15.** The top-view SEM image of a) μSi anode and b) μSi@LiSi anode after 100 cycles.

**Figure S16.** The cross-section SEM images and elemental mapping of a) μSi anode and b) μSi@LiSi anode before and after cycling.

**Figure S17**. XPS depth-profiling spectra of the Si anode after 100 cycles: a) Li 1s, b) P 2p, and c) S 2p.

**Figure S18**. XPS depth-profiling spectra of the μSi@LiSi anode after 100 cycles: a) Li 1s, b) P 2p, and c) S 2p.

**Table S1.** Comparison of long-term cycle stability of μSi@LiSi | NCM cell with reported ASSLIBs with areal capacity higher than 1 mAh cm^-2^.

| **No.** | **ASSBs**  **(cathode\|SE\|**  **anode)** | **T/°C** | **Capacity retention after 200 cycles/%** | **Cycle numbers** | **Current density/**  **mA cm^-2^** | **ICE/%** | **Reference** |
| --- | --- | --- | --- | --- | --- | --- | --- |
| 1 | NCM811\|LPSC\|Si-LPSC-C | 30 | 60 | 50 | 0.5 | 74.0 | Ref.5 |
| 2 | NCM811\|LPSC\|Si | 30 | 85 | 500 | 5 | 96.0 | Ref.6 |
| 3 | NCM811\|\|LPSC\|Li_4.4_Si-nSi | 25 | 83 | 400 | 0.48 | 77.3 | Ref.15 |
| 4 | LCO\|Li_3_InCl_6_/LPSC\|Si/Li_21_Si_5_ | 30 | 73 | 300 | 1.33 | 82.0 | Ref.16 |
| 5 | LCO\|LPSC\|Li_1_Si | 30 | 85 | 1000 | 5 | 95.7 | Ref.20 |
| 6 | NCM811\|LPSC\|PL-Si | 25 | 78 | 300 | 1.5 | 96.7 | Ref.22 |
| 7 | NCM83\|LPSC\|μSi@LiSi | 30 | 89 | 1000 | 1.6 | 82.1 | **This work** |
